# Supplementary material for: Structure-guided discovery of Otopetrin 1 inhibitors reveals druggable binding sites at the intrasubunit interface
Source: Nat Commun. 2025 Oct 23;16:9362. doi: 10.1038/s41467-025-64392-0 (PMC12549899; doi:10.1038/s41467-025-64392-0)

# Structure-Guided Discovery of Otopetrin 1 Inhibitors Reveals Druggable Binding Sites at the Intrasubunit Interface

Authors:

Batuujin Burendei[1†], Joshua P. Kaplan[2,3†], Gerardo M. Orellana[2], Emily R. Liman[2,3*], Stefano Forli[1*], and Andrew B. Ward[1*]

Affiliations:

1. Department of Integrative Structural and Computational Biology, The Scripps Research Institute, La Jolla, CA, 92037, USA
2. Section of Neurobiology, Department of Biological Sciences, University of Southern California, Los Angeles, CA, 90089, USA
3. Program in Neuroscience, University of Southern California, Los Angeles, CA, 90089, USA

Correspondence to: Emily R. Liman (liman@usc.edu), Stefano Forli (forli@scripps.edu), Andrew B. Ward (andrew@scripps.edu)

[†]These authors contributed equally: Batuujin Burendei, Joshua P. Kaplan
*These authors jointly supervised this work: Emily R. Liman, Stefano Forli, Andrew B. Ward

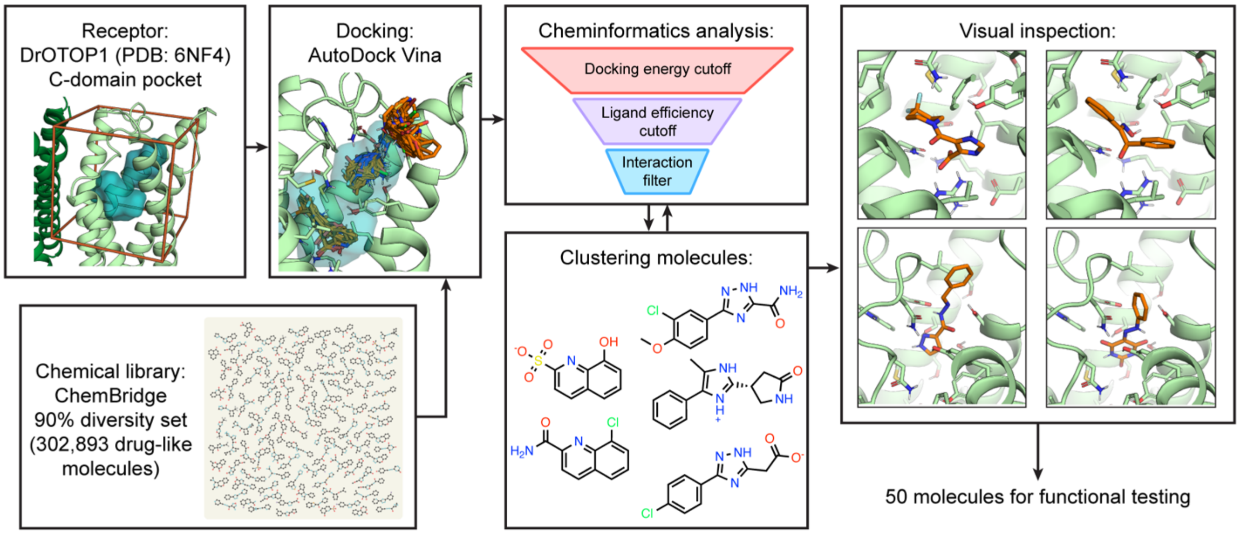


**Supplementary Figure 1.** Virtual screening on zebrafish OTOP1

Schematic summarizing the virtual screening pipeline, against the C-domain pocket of zebrafish OTOP1 (DrOTOP1). Cryo-EM structure of DrOTOP1 (PDB: 6NF4) is shown as cartoon, with the N-domain colored dark green and C-domain colored green. Docking box is shown as brown sticks. C-domain pocket is shown as blue transparent surface. Small molecules shown as sticks, colored orange.

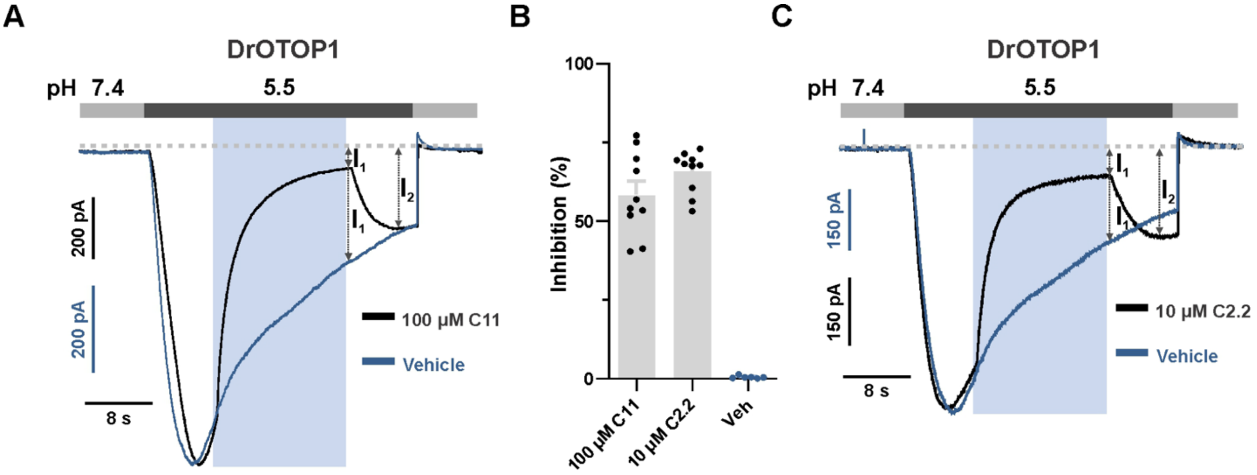


**Supplementary Figure 2.** Measurement of inhibition and vehicle control
**(A)** Representative traces of DrOTOP1 currents in response to pH 5.5 stimulation, followed by application of 100 μM C11 or vehicle control (0.3% DMSO), and washout with pH 5.5 alone. The 0.3% DMSO was used as a control for all DMSO concentrations. **(B)** Quantification of inhibition by C11, C2.2, and vehicle (0.3% DMSO). Inhibition was calculated as $((I_{2peak}-I_1) / I_{2peak}) * 100$, where $I_1$ is the current immediately before wash off and $I_{2peak}$ is the maximum recovered current. No inhibition was detected with 0.3% DMSO. **(C)** Same as (A), but with 10 μM C2.2. Source data are provided as a Source Data file.

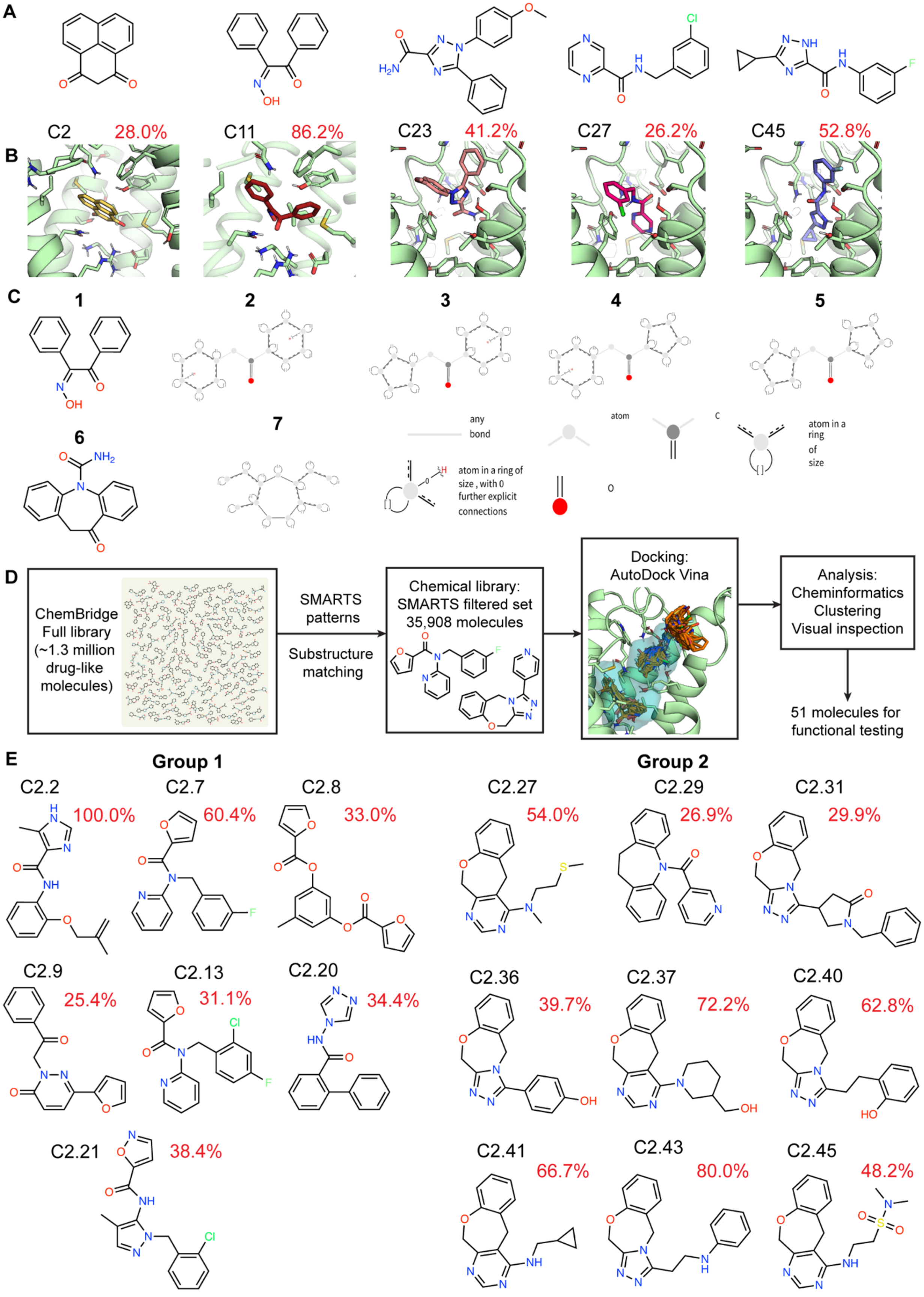
A
B
C2 28.0%
C11 86.2%
C23 41.2%
C27 26.2%
C45 52.8%
C
1
2
3
4
5
6
7
any bond
atom
C
atom in a ring of size
atom in a ring of size, with 0 further explicit connections
O
D
ChemBridge Full library (~1.3 million drug-like molecules)
SMARTS patterns
Substructure matching
Chemical library: SMARTS filtered set 35,908 molecules
Docking: AutoDock Vina
Analysis: Cheminformatics Clustering Visual inspection
51 molecules for functional testing
E
Group 1
Group 2
C2.2 100.0%
C2.7 60.4%
C2.8 33.0%
C2.9 25.4%
C2.13 31.1%
C2.20 34.4%
C2.21 38.4%
C2.27 54.0%
C2.29 26.9%
C2.31 29.9%
C2.36 39.7%
C2.37 72.2%
C2.40 62.8%
C2.41 66.7%
C2.43 80.0%
C2.45 48.2%

**Supplementary Figure 3.** Functional inhibitors identified from virtual screening
**(A)** 2D chemical structures and **(B)** docked poses of inhibitors identified from the first round of screening. DrOTOP1_apo model (PDB: 6NF4) is shown in green sticks and cartoon. Degrees of % inhibition at 200 μM for each inhibitor are shown in red. **(C)** 2D chemical structures of C11 (**1**), SMARTS patterns designed based on C11 (**2 – 5),** oxcarbazepine (**6**), and SMARTS pattern designed based on oxcarbazepine (**7**) are shown, along with the SMARTS legend. **(D)** Schematic summarizing the second round of virtual screening. **(E)** 2D chemical structures of inhibitors identified from the second round of screening, separated into two groups. Degrees of % inhibition at 200 μM for each inhibitor are shown in red.

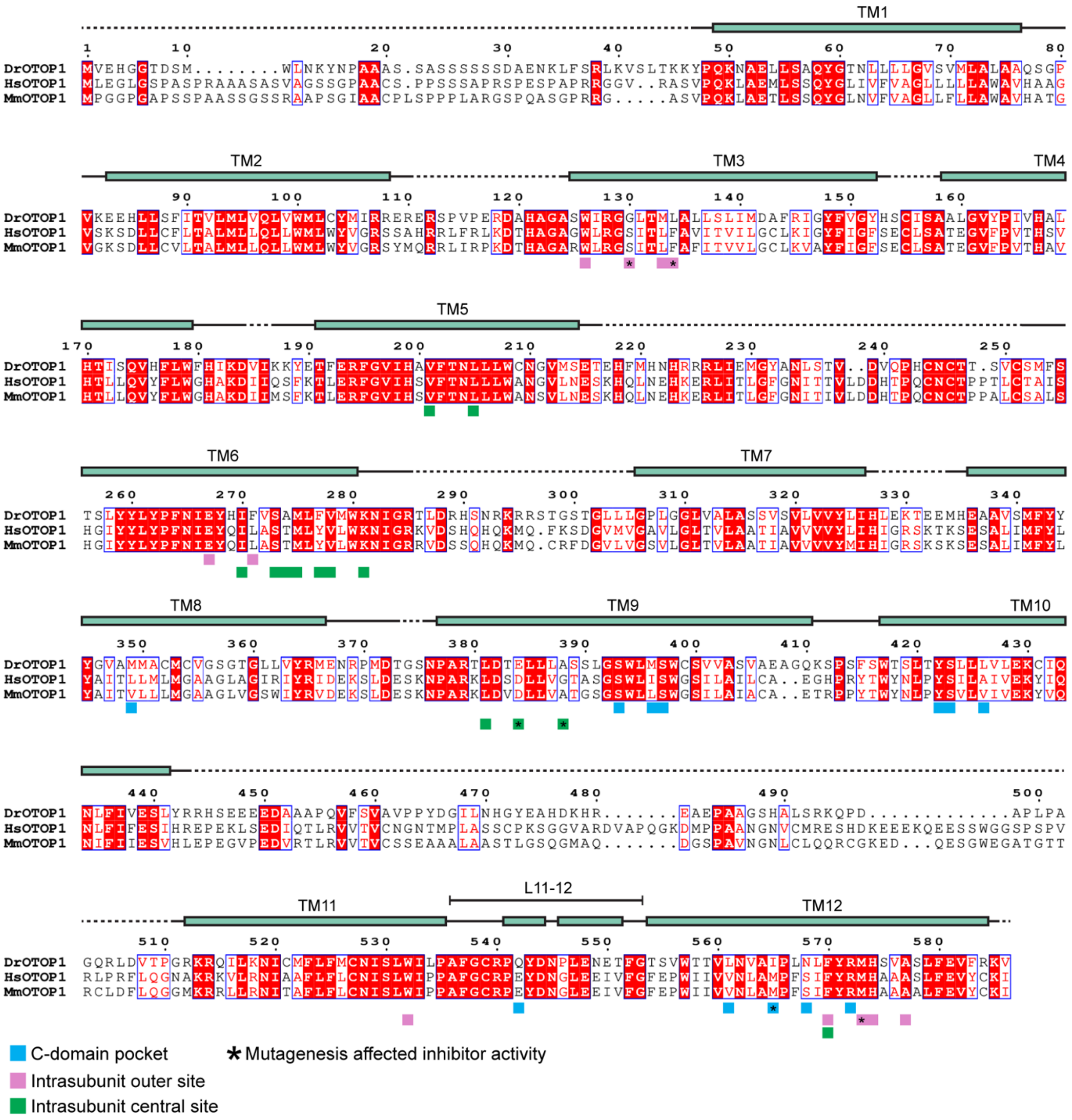


**Supplementary Figure 4.** Topology and sequence alignment of zebrafish OTOP1 and mammalian OTOP1

Sequence alignment chart of zebrafish OTOP1 (DrOTOP1, Uniprot ID: Q7ZWK8), human OTOP1 (hOTOP1, Uniprot ID: Q7RTM1), and mouse OTOP1 (mOTOP1, Uniprot ID: Q80VM9). Identical matches are colored solid red, and similar residues are displayed in boxes. Transmembrane helices of DrOTOP1 are indicated as green rectangles, modeled linkers as solid lines, and unmodelled residues as dotted lines. The linker L11-12 loop contains short alpha helical elements and is indicated. Residues lining each virtual screening pocket are indicated using colored squares, blue for C-domain pocket, pink for intrasubunit interface outer site, and green for intrasubunit central site. Residues that were mutated and had an effect on inhibitor activity are annotated with asterisks, inside the squares.

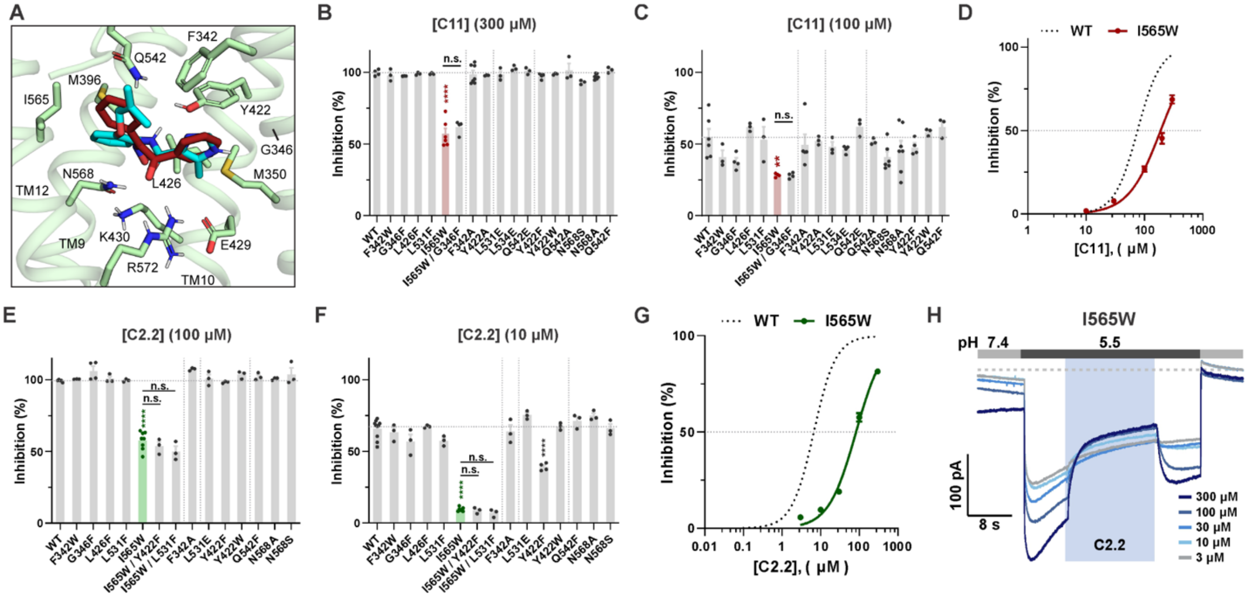


I

| Pocket | Mutation | Desired effect |
|---|---|---|
| C-domain | F342W | Bulky sidechain introduction for steric hindrance |
| | G346F | |
| | L426F | |
| | L531F | |
| | I565W | |
| | I565W / G346F | |
| | I565W / Y422F | |
| | I565W / L531F | |
| | F342A | Removal of pi-stack partner |
| | Y422A | |
| | L531E | Negative charge introduction |
| | L534E | |
| | Q542E | |
| | Y422F | Removal of H-bond partner |
| | Y422W | |
| | Q542A | |
| | N568A | |
| | N568S | |
| | Q542F | Removal of charge and bulky sidechain introduction |

**Supplementary Figure 5.** Mutagenesis of C-domain pocket lining residues and effects on inhibition by C11 and C2.2

**(A)** Docked poses of C11 (dark red) and C2.2 (cyan) in the C-domain pocket superimposed, with the DrOTOP1_apo model (PDB: 6NF4) shown in sticks and cartoons and the pocket-lining residues labeled. **(B, C)** Data (mean ± SEM) measuring inhibition of C11 in HEK-293 cells expressing DrOTOP1 mutants targeting the C-domain pocket. Mutants are grouped by expected effects (dotted lines on the X-axis, as in (I)). Mutants were tested against C11 at a saturating concentration (B) (300 μM) and near the $IC_{50}$ (C) (100 μM). **(D)** Dose-response inhibition by C11 for WT (black) and I565W (red). WT data from Fig. 2C. Data were fit to the Hill equation to determine $IC_{50}$ and Hill coefficients (mean ± SEM, n = 9). I565W: $IC_{50}$ = 190.1 μM, h = 1.57. **(E, F)** Data measuring inhibition of C2.2 in HEK-293 cells expressing a subset of the same mutations made in (B, C), exposed to either 100 μM (E) or 10 μM (F). (n ≥ 3 for each mutation tested). **(G)** Dose-response inhibition by C2.2 for WT (black) and I565W (green). Data were fit to the Hill equation to determine $IC_{50}$ and Hill coefficient (mean ± SEM, n = 8). I565W: $IC_{50}$ = 82.9 μM, h = 1.22. WT data from Fig. 2C. **(H)** Representative traces of I565W showing dose-dependence by C2.2 over a range of 300-3 μM as measured in (G). **(I)** Table summarizing mutations tested in the C-domain pocket. All data are presented as mean ± SEM. All experiments performed by holding cells at $V_m$ = -80 mV. Significance was determined by one-way ANOVAs with Dunnett's correction

(single mutants vs. WT in B, C, E, F) and unpaired two-tailed t-tests (double mutants vs. I565W in B, C, E, F). **P* < 0.05, ***P* < 0.01, ****P* < .001, *****P* < 0.0001 for all statistical tests. Source data are provided as a Source Data file.

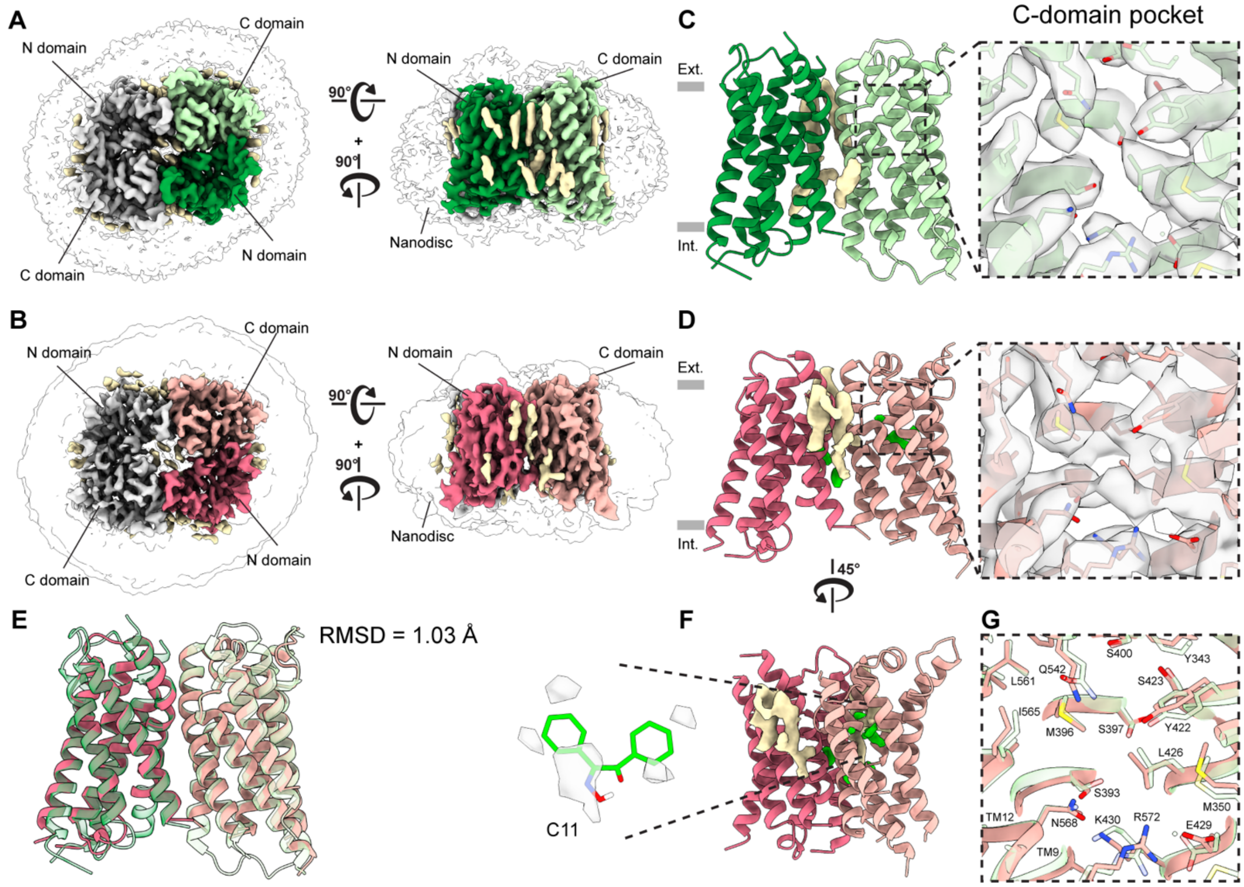


**Supplementary Figure 6.** Cryo-EM structure of DrOTOP1_apo and DrOTOP1_C11
**(A)** Top (left) and side (right) views of DrOTOP1_apo cryo-EM structure (EMDB: 9360), with one monomer colored dark green (N-domain) and light green (C-domain), the other monomer colored light grey, bound-lipids colored tan and the nanodisc micelle shown as transparent surface. **(B)** Side (top) and top (bottom) views of cryo-EM structures of DrOTOP1 in complex with C11 (DrOTOP1_C11), with one monomer colored dark red (N-domain) and light salmon (C-domain) and the other monomer colored light grey, bound-lipids colored tan, potential small molecules colored green, and the nanodisc micelle shown as transparent surface. **(C)** Side view of DrOTOP1_apo model (PDB: 6NF4), with density for bound lipids or inhibitors shown. Inset shows the C-domain pocket with the cryo-EM map shown as grey transparent surface. **(D)** Side view of the DrOTOP1_C11 model, with density for bound lipids and small molecules shown. Inset shows the C-domain pocket with the cryo-EM map shown as grey transparent surface. **(E)** Side view of alignment of DrOTOP1_C11 and DrOTOP1_apo (transparent), with the global RMSD (across all pairs) value reported on the right. **(F)** 45º rotated side view from (D) shows the C-domain pocket, with the inset showing non-protein density found within the pocket as transparent surfaces, with the inhibitor C11 (green) attempted to be modeled in, shown as sticks. **(G)** View of the C-domain pocket with DrOTOP1_apo (transparent) and DrOTOP1_C11 aligned and superimposed, with pocket lining residues labeled and shown as sticks, and backbones shown as cartoons to highlight conformational differences or the lack thereof.

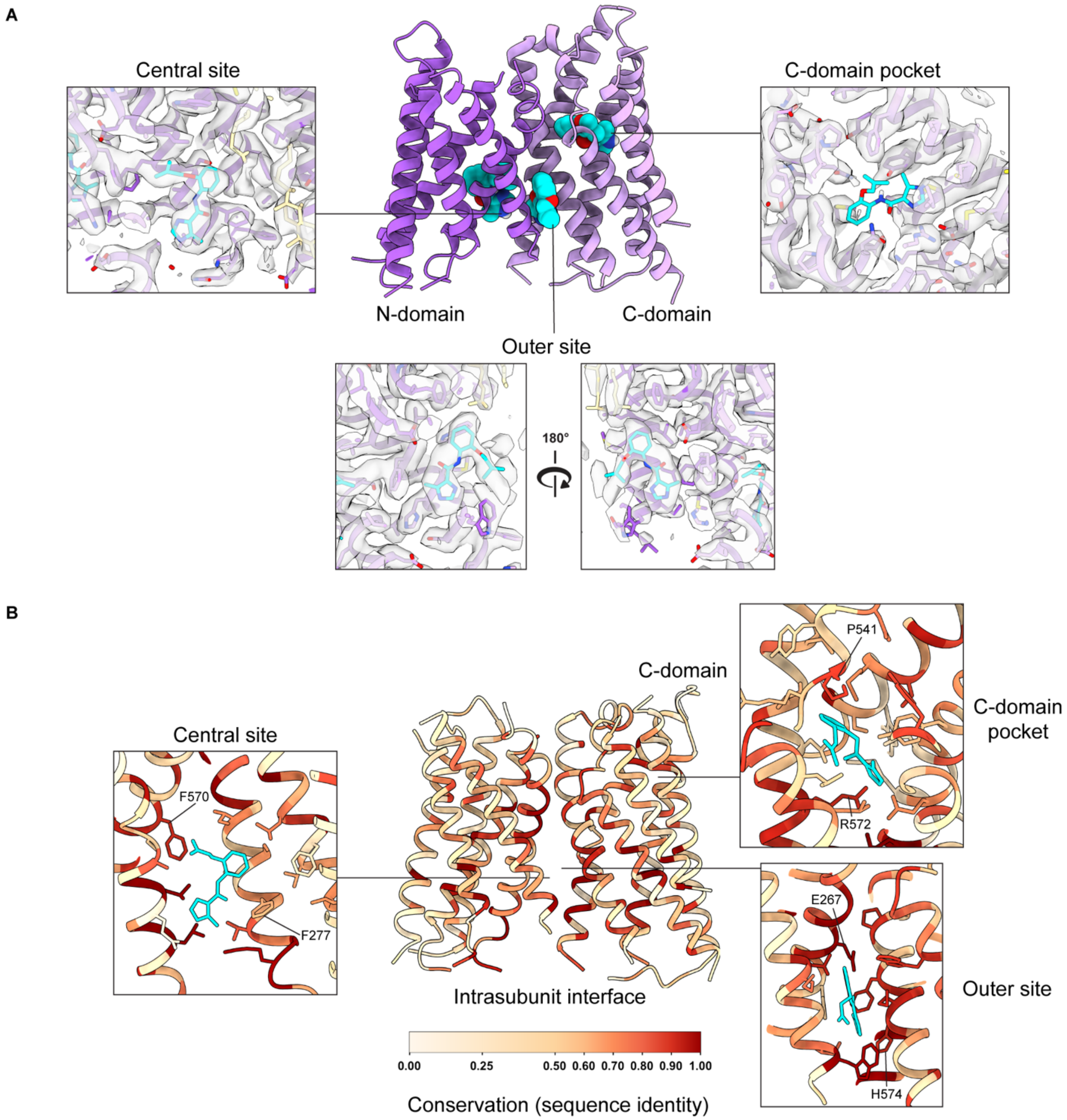


**Supplementary Figure 7.** Sequence conservation of Otopetrins in all considered binding sites for inhibitors

**(A)** Side view of DrOTOP1_C2.2 model shown as cartoon and colored purple (N-domain) and plum-pink (C-domain), shown as cartoon and four copies of C2.2 models shown as cyan spheres. Insets show zoom-ins on the central site, C-domain pocket, and outer site (two views), in clockwise from the left, with the cryo-EM map shown as transparent grey surface. C2.2 models (cyan), bound cholesterol or CHS (tan), and interacting residues are shown as sticks. C2.2 models in the C-domain pocket are not included in the final model, due to the lack of density in the region.

**(B)** Side view of DrOTOP1_C2.2 model shown as cartoon and colored with sequence identity fraction to highlight evolutionarily conserved regions across 58 vertebrate Otopetrin sequences. Insets show zoom-ins on the central site, C-domain pocket, and outer site, in clockwise from the

left. Modelled inhibitor C2.2 models (cyan) and interacting residues are shown as sticks, with select residues labeled. Color map unit is in fractions.

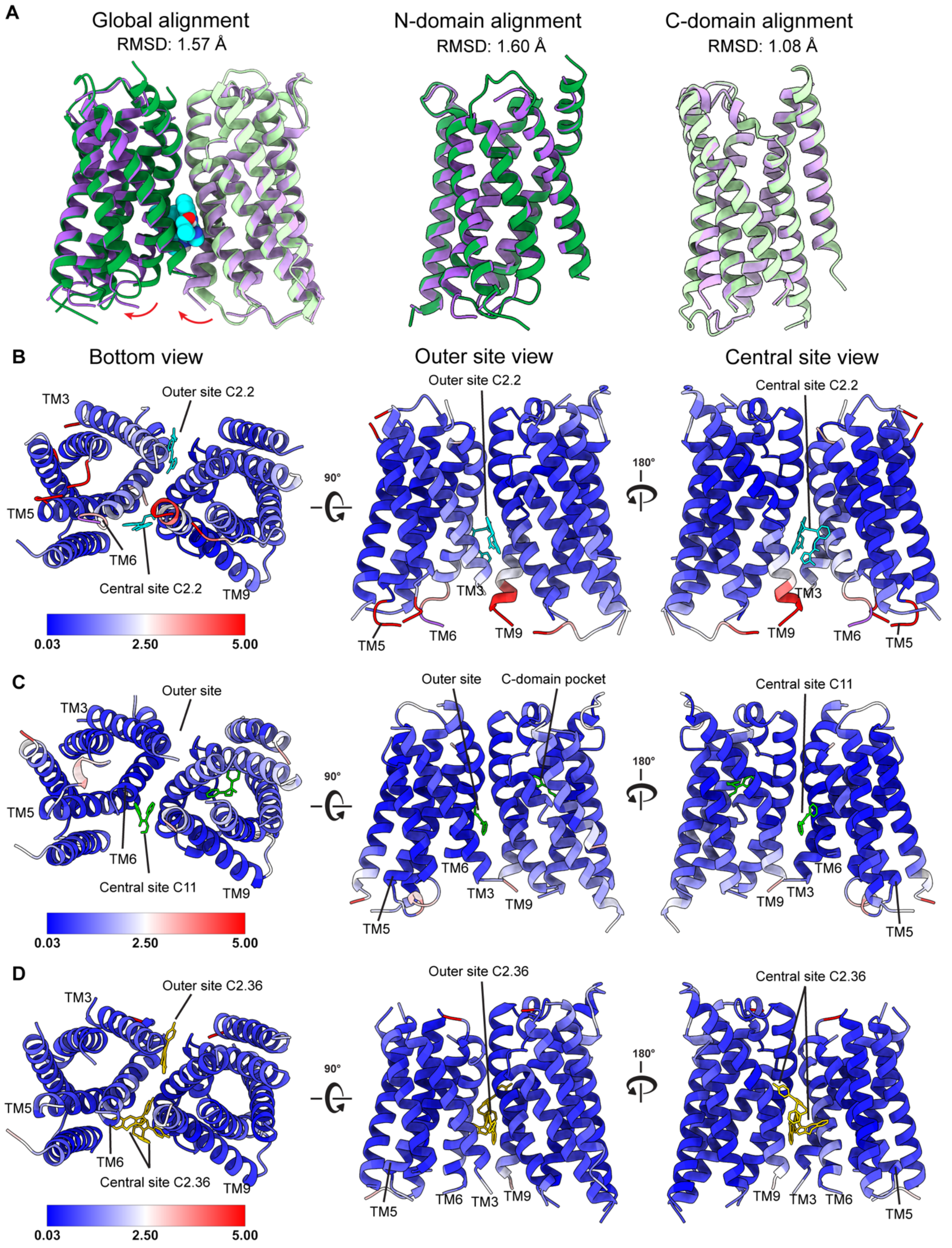


**Supplementary Figure 8.** Conformational changes in DrOTOP1-inhibitor complex cryo-EM structures

**(A)** Global (left), N-domain only (middle), and C-domain only (right) alignment and superimposition of DrOTOP1_C2.2 against DrOTOP1_apo, with calculated RMSD (across all pairs) reported for each. Key conformational changes are indicated in red arrows. Bound inhibitor C2.2 molecules are shown as cyan spheres. Models of DrOTOP1_C2.2 **(B)**, DrOTOP1_C11 **(C)**, and DrOTOP1_C2.36 **(D)** colored according to Cα RMSD values between their respective structures and DrOTOP1_apo, shown in three views. Inhibitor molecules are drawn as sticks, C2.2 in cyan, C11 in green, and C2.36 in gold, and the color bar unit is (Å). All RMSD calculations were made with the "matchmaker" command in UCSF ChimeraX.

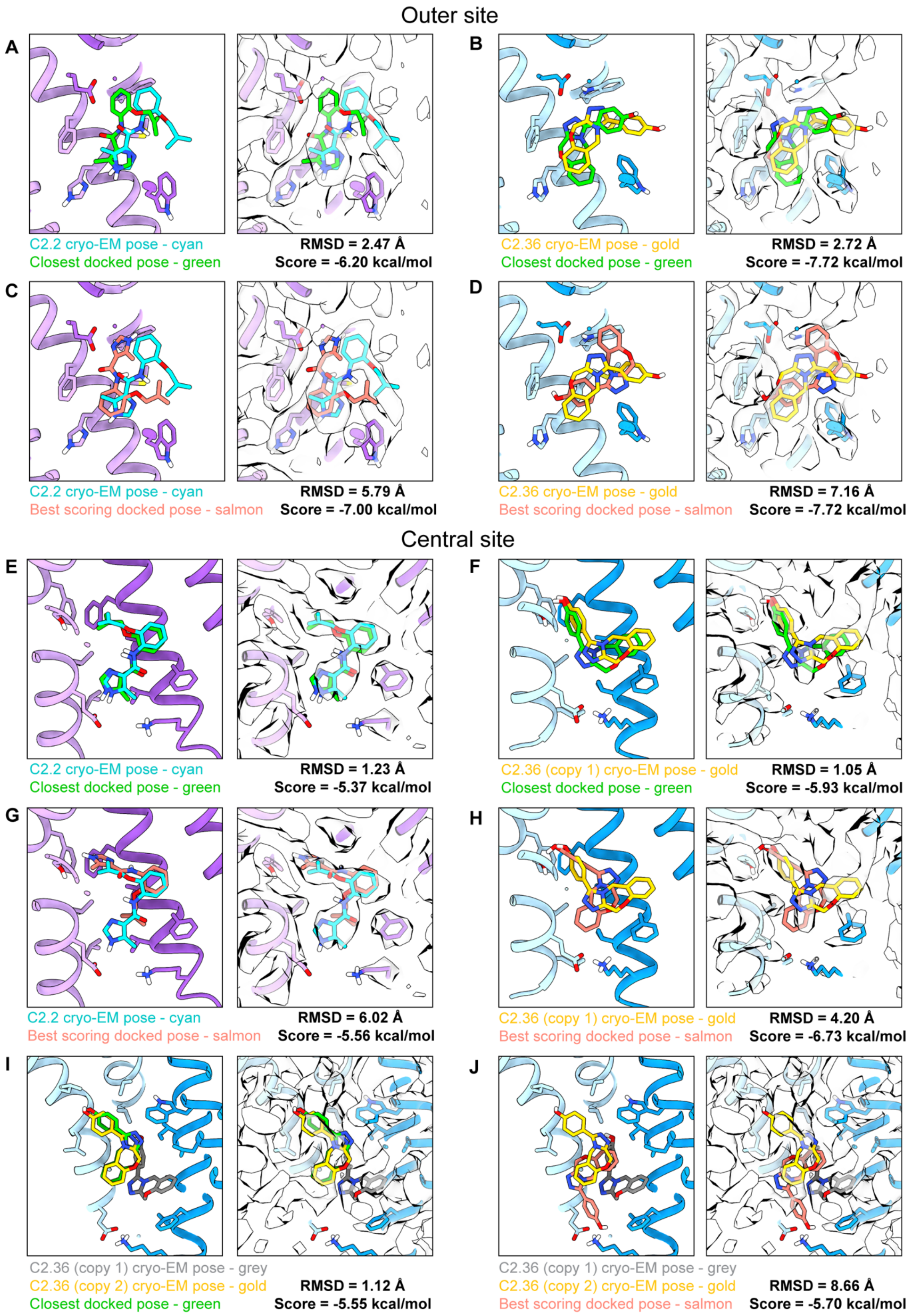
Outer site
A
C2.2 cryo-EM pose - cyan
Closest docked pose - green
RMSD = 2.47 Å
Score = -6.20 kcal/mol
B
C2.36 cryo-EM pose - gold
Closest docked pose - green
RMSD = 2.72 Å
Score = -7.72 kcal/mol
C
C2.2 cryo-EM pose - cyan
Best scoring docked pose - salmon
RMSD = 5.79 Å
Score = -7.00 kcal/mol
D
C2.36 cryo-EM pose - gold
Best scoring docked pose - salmon
RMSD = 7.16 Å
Score = -7.72 kcal/mol
Central site
E
C2.2 cryo-EM pose - cyan
Closest docked pose - green
RMSD = 1.23 Å
Score = -5.37 kcal/mol
F
C2.36 (copy 1) cryo-EM pose - gold
Closest docked pose - green
RMSD = 1.05 Å
Score = -5.93 kcal/mol
G
C2.2 cryo-EM pose - cyan
Best scoring docked pose - salmon
RMSD = 6.02 Å
Score = -5.56 kcal/mol
H
C2.36 (copy 1) cryo-EM pose - gold
Best scoring docked pose - salmon
RMSD = 4.20 Å
Score = -6.73 kcal/mol
I
C2.36 (copy 1) cryo-EM pose - grey
C2.36 (copy 2) cryo-EM pose - gold
Closest docked pose - green
RMSD = 1.12 Å
Score = -5.55 kcal/mol
J
C2.36 (copy 1) cryo-EM pose - grey
C2.36 (copy 2) cryo-EM pose - gold
Best scoring docked pose - salmon
RMSD = 8.66 Å
Score = -5.70 kcal/mol

**Supplementary Figure 9.** Docking of C2.2 and C2.36 into intrasubunit sites
On the left, closest docked poses (green) of C2.2 **(A)** and C2.36 **(B)** in the intrasubunit outer site, with the corresponding cryo-EM structures (DrOTOP1_C2.2 – pink / purple, DrOTOP1_C2.36 – light blue / blue) and cryo-EM modelled poses shown (C2.2 – cyan, C2.36 – gold). On the right, corresponding cryo-EM density maps are overlayed as transparent surfaces. Similarly, the best scoring docked poses for C2.2 **(C)** and C2.36 **(D)** are shown. **(E - H)** The equivalent comparisons as in (A) – (D), respectively, for C2.2 and C2.36 docked into the central sites. **(I, J)** Similar analysis as (F) and (H) for the second copy of C2.36 in the central site. All RMSD calculations were made with the “rmsd” command in UCSF ChimeraX.

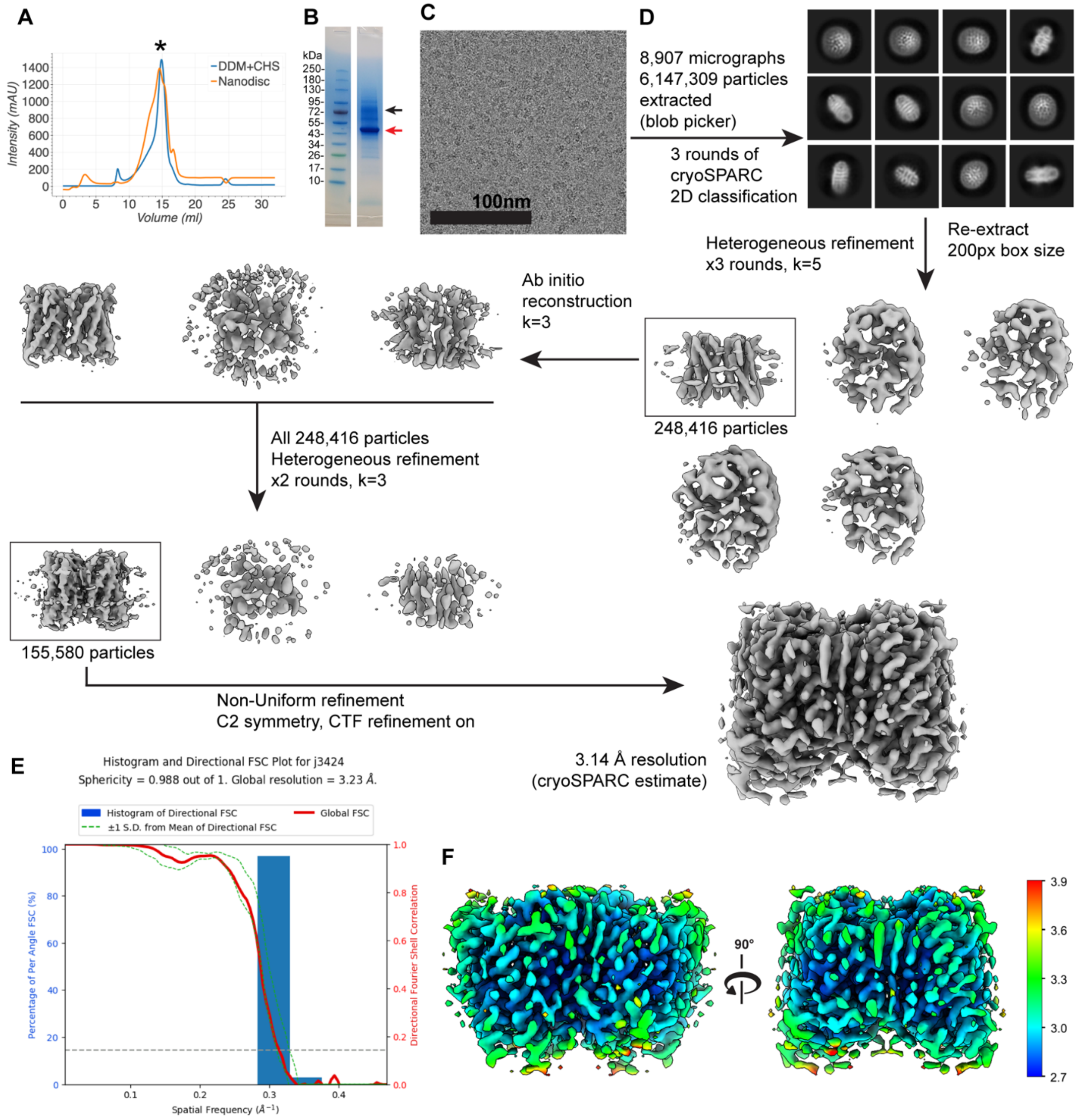


**Supplementary Figure 10.** Cryo-EM data processing for DrOTOP1_C2.2
**(A)** Size-exclusion chromatography (SEC) traces for DrOTOP1 purification in DDM+CHS and nanodisc. The data for the DDM+CHS trace is offset by -300 in the y-axis, for a clearer comparison with the nanodisc trace. The peaks corresponding to DrOTOP1 are denoted with * and were collected. **(B)** SDS-PAGE analysis of DrOTOP1 peak fraction, with bands corresponding to DrOTOP1 (black arrow) and MSP2N2 (red arrow) are denoted. The marker lane and sample lane were run on the same gel and were re-arranged to be adjacent. **(C)** Exemplar micrograph from cryo-EM image collection. Scale bar is 100nm. **(D)** Summary of cryo-EM processing steps taken to obtain the final reconstructed map. **(E)** 3D-FSC graph of final map of DrOTOP1_C2.2. **(F)** Final map colored according to estimated local resolution. Color bar unit is (Å). Source data are provided as a Source Data file and at the end of the Supplementary Information file.

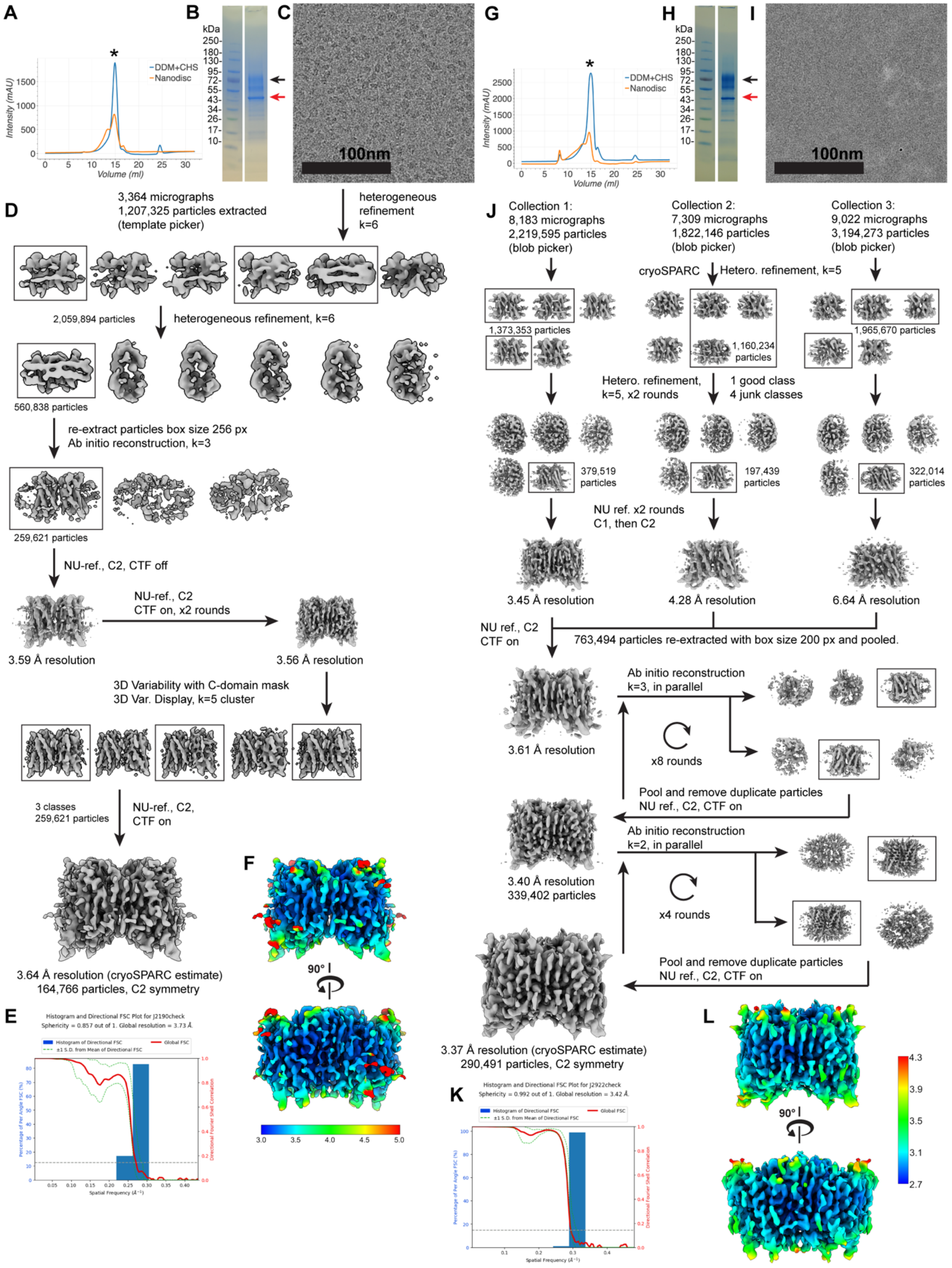


**Supplementary Figure 11.** Cryo-EM data processing for DrOTOP1_C11 and DrOTOP1_C2.36

SEC traces for DrOTOP1 purification in DDM+CHS and nanodisc for DrOTOP1_C11 **(A)** and DrOTOP1_C2.36 **(G)** samples. The peaks corresponding to DrOTOP1 are denoted with * and were collected. SDS-PAGE analysis of DrOTOP1 peak fractions, with bands corresponding to DrOTOP1 (black arrow) and MSP2N2 (red arrow) are denoted for DrOTOP1_C11 **(B)** and DrOTOP1_C2.36 **(H)**. The marker lanes and sample lanes for were run on the same gels and were re-arranged to be adjacent. Exemplar micrographs from cryo-EM image collections for DrOTOP1_C11 **(C)** and DrOTOP1_C2.36 **(I)**. Scale bars are 100nm. Summaries of cryo-EM processing steps taken to obtain final reconstructed maps for DrOTOP1_C11 **(D)** and DrOTOP1_C2.36 **(J)**. 3D-FSC graphs of final maps of DrOTOP1_C11 **(E)** and DrOTOP1_C2.36 **(K)**. Final maps colored according to estimated local resolutions for DrOTOP1_C11 **(F)** and DrOTOP1_C2.36 **(L)**. Color bar units are (Å). Source data are provided as a Source Data file and at the end of the Supplementary Information file.

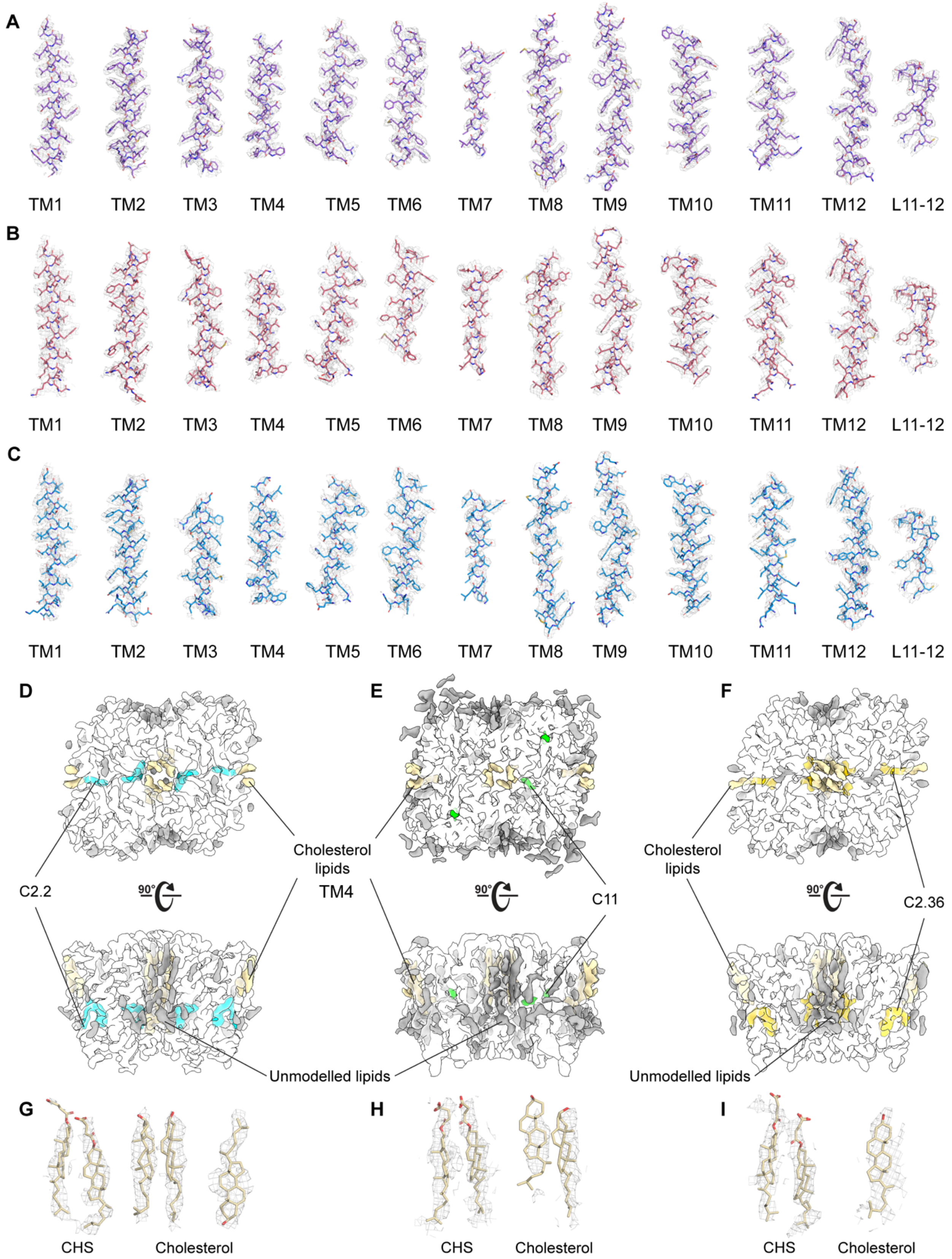


**Supplementary Figure 12.** Fit of built models to cryo-EM map density DrOTOP1_C2.2,

DrOTOP1_C11, and DrOTOP1_C2.36

Isolated models of TM helices 1 to 12 and loop 11-12 (L11-12) of DrOTOP1_C2.2 **(A)**, DrOTOP1_C11 **(B)**, and DrOTOP1_C2.36 **(C)** cryo-EM structures shown as sticks, with the map densities shown at 4 $\sigma$, 5 $\sigma$, and 4 $\sigma$ in grey mesh, respectively. Top (top) and side (bottom) views of DrOTOP1_C2.2 **(D)**, DrOTOP1_C11 **(E)**, and DrOTOP1_C2.36 **(F)** cryo-EM maps shown as surfaces, with protein density colored transparent white, bound inhibitor density colored cyan (C2.2), green (C11), or gold (C2.36), modelled bound cholesterol or CHS density colored tan, and unmodelled bound lipids colored grey. Fit of modelled bound cholesterol of CHS molecules into map densities for DrOTOP1_C2.2 **(G)**, DrOTOP1_C11 **(H)**, and DrOTOP1_C2.36 **(I)**, with maps shown at the same $\sigma$ levels as (A), (B), and (C), respectively.

A

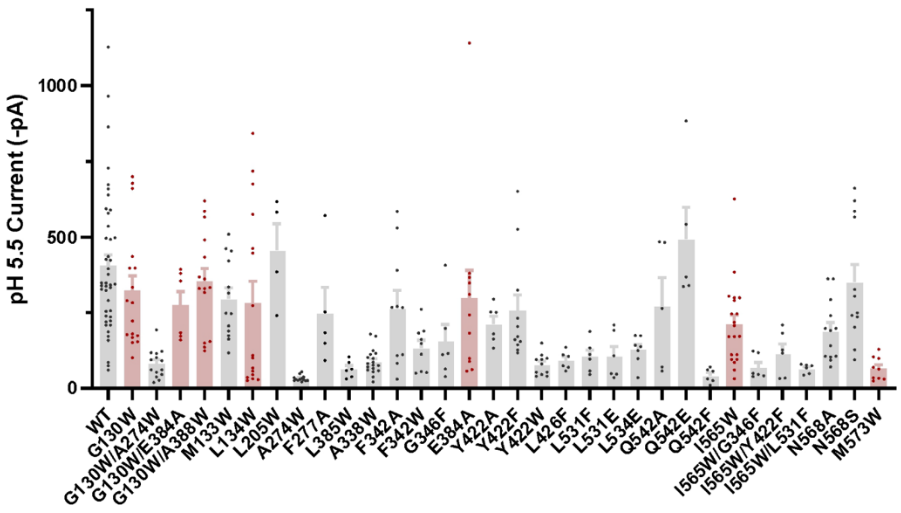


B

| Dunnett's multiple comparisons test | Summary | Adjusted P Value |
|---|---|---|
| WT vs. G130W | ns | 0.8972 |
| WT vs. G130W/A274W | **** | <0.0001 |
| WT vs. G130W/E384A | ns | 0.8355 |
| WT vs. G130W/A388W | ns | >0.9999 |
| WT vs. M133W | ns | 0.607 |
| WT vs. L134W | ns | 0.238 |
| WT vs. L205W | ns | >0.9999 |
| WT vs. A274W | **** | <0.0001 |
| WT vs. F277A | ns | 0.7005 |
| WT vs. L385W | **** | <0.0001 |
| WT vs. A338W | **** | <0.0001 |
| WT vs. F342A | ns | 0.3271 |
| WT vs. F342W | *** | 0.0003 |
| WT vs. G346F | * | 0.0152 |
| WT vs. E384A | ns | 0.7603 |
| WT vs. Y422A | ns | 0.179 |
| WT vs. Y422F | ns | 0.1796 |
| WT vs. Y422W | **** | <0.0001 |
| WT vs. L426F | *** | 0.0004 |
| WT vs. L531E | ** | 0.001 |
| WT vs. L531F | *** | 0.001 |
| WT vs. L534E | ** | 0.0011 |
| WT vs. Q542A | ns | 0.9017 |
| WT vs. Q542E | ns | 0.9996 |
| WT vs. Q542F | **** | <0.0001 |
| WT vs. I565W | *** | 0.0005 |
| WT vs. I565W/G346F | *** | 0.0001 |
| WT vs. I565W/Y422F | ** | 0.0019 |
| WT vs. I565W/L531F | **** | <0.0001 |
| WT vs. N568A | ** | 0.0012 |
| WT vs. N568S | ns | >0.9999 |
| WT vs. M573W | **** | <0.0001 |

**Supplementary Figure 13.** Current magnitudes of all mutants tested in response to a pH 5.5 stimulus

**(A)** Data measuring current magnitudes in response to extracellular acidification (pH 5.5) of all mutants tested from experiments as in (Fig. 5D, H, L, N) and Supplementary Figure 4 B, C, E, F) (n ≥ 5 for each construct). Mutants in red (G130W, G130W/A388W, G130W/E348A, L134W, E384A, I565W, and M573W) reduced inhibition by C2.2. **(B)** Ordinary one-way ANOVA corrected for multiple comparisons with Dunnett's correction with summary of significance and adjusted p-values for each construct. *$P < 0.05$, **$P < 0.01$, ***$P < .001$, ****$P < 0.0001$. All data are presented as mean ± SEM. All experiments performed by holding cells at $V_m$ = -80 mV. Source data are provided as a Source Data file.

**Supplementary Table 1.** Chemicals tested for DrOTOP1 inhibitor screening

| Nickname | ChemBridge ID | SMILES |
|---|---|---|
| Chemicals of interest | | |
| C11 (BMO) | 5572857 | c1ccc(cc1)/C(=N/O)/C(=O)c2ccccc2 |
| C2.2 | 15302368 | C=C(C)COc1ccccc1NC(=O)c1nc[nH]c1C |
| C2.36 | 68710218 | Oc1ccc(-c2nnc3n2Cc2ccccc2OC3)cc1 |
| First round of screening | | |
| C1 | 5175100 | c1cc2ccc(nc2c(c1)O)S(=O)(=O)[O-] |
| C2 | 5175175 | c1cc2cccc3c2c(c1)C(=O)CC3=O |
| C3 | 5855260 | c1ccc(cc1)/C=N/NC(=O)c2cc[nH]n2 |
| C4 | 9308998 | c1ccc2c(c1)c3c(cn2)C(=O)OCCO3 |
| C5 | 5380478 | c1cnccc1/C=N/NC(=O)c2c(non2)N |
| C6 | 7648452 | Cc1cc(=O)c2c(o1)c3ccccc3oc2=O |
| C7 | 6175236 | Cc1c(c(n[nH]1)O)/N=N/c2ccccc2C#N |
| C8 | 7062980 | c1ccc2c(c1)c(c(c(n2)O)C(=O)[O-])C(=O)[O-] |
| C9 | 5491209 | Cc1ccc(cc1)C(=O)O/N=C(/c2c(non2)N)\N |
| C10 | 5566026 | c1cc(ccc1/C=N/Nc2cn[nH]c(=O)n2)O |
| C12 | 6060170 | Cc1cccc(c1)c2nc(on2)c3ccco3 |
| C13 | 9257635 | Cc1nc(co1)C(=O)NCc2ccc3c(c2)OCO3 |
| C14 | 5905516 | c1cc(ncc1Br)NC(=O)CSc2[nH]ncn2 |
| C15 | 5611103 | CC(=NNC(=O)C(=O)NCc1ccccc1)C |
| C16 | 7225880 | CC(C)(C)c1ccc(cc1)C(=O)Nc2[n-]nnn2 |
| C17 | 9192355 | c1ccc(cc1)c2[nH]nc(n2)Nc3ncccn3 |
| C18 | 7961841 | Cc1cccc(c1)c2nc(on2)CCC(=O)NC |
| C19 | 7921156 | [H]/N=C(/NCCc1c[nH]c2c1cccc2)\NC(=[NH2+])N |
| C20 | 5142100 | COc1ccc(cc1)NN=C2C(=O)NC(=O)NC2=O |
| C21 | 9281699 | c1cc(ccc1c2nc([nH]n2)c3cnccn3)Cl |
| C22 | 9244891 | c1cc(ccc1c2nc([nH]n2)CC(=O)[O-])Cl |
| C23 | 9118364 | COc1ccc(cc1)n2c(nc(n2)C(=O)N)c3ccccc3 |
| C24 | 19833772 | c1cc(oc1C[NH+]2CCC3(C2)CC[NH2+]CC3)c4ccn[nH]4 |
| C25 | 5315914 | c1(nc(on1)C(F)(F)F)c2nc(on2)C(F)(F)F |
| C26 | 9192269 | c1ccc2c(c1)[nH]c([nH+]2)NCc3ccccn3 |
| C27 | 7943917 | c1cc(cc(c1)Cl)CNC(=O)c2cnccn2 |
| C28 | 5324731 | c1cc(cc(c1)Br)/C=N/Nc2c(=O)[nH]c(=O)[nH]n2 |
| C29 | 5790139 | Cc1ccc(c2c1nsn2)/C=N/Nc3[n-]nnn3 |
| C30 | 6046235 | c1ccc2c(c1)c(c[nH]2)/C=N/Nc3[nH]ncc(=O)n3 |
| C31 | 9253306 | c1ccc2c(c1)nc(s2)C(=O)Nc3ccccn3 |
| C32 | 98258293 | Cc1ccc(nn1)c2nc(on2)[C@H]3CCC[NH2+]3 |

| | | |
|---|---|---|
| C33 | 66878413 | Cc1nc(on1)c2ccc(o2)c3ccccc3Cl |
| C34 | 33468535 | COCc1nc(on1)CNC(=O)c2ccccc2 |
| C35 | 9034869 | c1ccnc(c1)c2[nH]nc(n2)c3cccc(c3)Br |
| C36 | 68242534 | Cc1ccc(cn1)[C@@H](C)NC(=O)Nc2[n-]nnn2 |
| C37 | 49081967 | CCc1nnc(o1)c2[nH]c3c(n2)C(=O)NCCC3 |
| C38 | 64024501 | Cc1csc(n1)NC(=O)c2[n-]nc(n2)C3CC3 |
| C39 | 73048518 | Cc1nc(on1)c2csc(n2)Cc3ccccc3 |
| C40 | 26754303 | Cc1c([nH+]c([nH]1)[C@@H]2CC(=O)NC2)c3ccccc3 |
| C41 | 31000082 | c1[nH]c(c(n1)C(=O)[O-])C(=O)N2CCC(CC2)(F)F |
| C42 | 49266714 | COCc1nc(no1)c2[nH]c3c(n2)C(=O)NCCC3 |
| C43 | 59123264 | Cc1nc([nH]n1)c2ccc(o2)C[NH+]3CCC(CC3)C |
| C44 | 57626143 | Cc1nc([nH]n1)CNC(=O)c2cnc3ccccc3n2 |
| C45 | 77727795 | c1cc(cc(c1)F)NC(=O)c2[nH]nc(n2)C3CC3 |
| C46 | 72294446 | c1cc2ccc(nc2c(c1)Cl)C(=O)N |
| C47 | 5174634 | c1ccc(cc1)NN=C2C(=O)NC(=O)NC2=O |
| C48 | 55766621 | Cc1cnc(cn1)c2[nH]c3c(n2)C(=O)NCCC3 |
| C49 | 73936204 | COc1ccc(cc1Cl)c2nc([nH]n2)C(=O)N |
| C50 | 67084589 | Cc1ccccc1[C@@]2(CCN(C2)CC(F)F)O |
| Second round of screening | | |
| C2.1 | 6439558 | Cc1nn2c3ccccc3n(CC(=O)c3ccccc3)c2nc1=O |
| C2.3 | 9065258 | CCC(=O)Nc1ccccc1OC(=O)c1ccccc1F |
| C2.4 | 9252815 | COc1cccc(N2Cc3n[nH]c(=O)n3C2=O)c1 |
| C2.5 | 7994652 | CS(=O)(=O)c1ncc(Cl)c(C(=O)Oc2ccccc2Cl)n1 |
| C2.6 | 9274834 | O=C(Nc1ccccc1-c1nc(Cc2ccccc2)no1)c1ccco1 |
| C2.7 | 9337394 | O=C(c1ccco1)N(Cc1cccc(F)c1)c1ccccn1 |
| C2.8 | 5106578 | Cc1cc(OC(=O)c2ccco2)cc(OC(=O)c2ccco2)c1 |
| C2.9 | 9343175 | O=C(Cn1nc(-c2ccco2)ccc1=O)c1ccccc1 |
| C2.10 | 7895373 | O=C(Oc1ccccc1)c1cccc(COc2ccccc2Cl)c1 |
| C2.11 | 9038676 | O=C(Nc1nc[nH]n1)c1cccc(OCc2ccccc2)c1 |
| C2.12 | 6552073 | Cc1ccccc1OC(=O)c1cccc(C(=O)Oc2ccccc2C)c1 |
| C2.13 | 9335653 | O=C(c1ccco1)N(Cc1ccc(F)cc1Cl)c1ccccn1 |
| C2.14 | 5172733 | O=C(O)c1cccc2c(=O)oc3ccccc3c12 |
| C2.15 | 5806283 | Cc1nc2c(=O)oc3ccccc3c2n1-c1ccccc1 |
| C2.16 | 7654950 | Nn1c(=O)c2ccccc2n2c(SCc3ccccc3)nnc12 |
| C2.17 | 85624791 | Cc1cnn(C(C)C2CC2)c1NC(=O)c1cn[nH]c1-n1cnnn1 |
| C2.18 | 6810466 | O=C(Nc1ccccc1C(F)(F)F)c1nnc[nH]1 |
| C2.19 | 5133421 | O=C(c1ccccc1)C1(c2ccccc2)COCO1 |
| C2.20 | 7714219 | O=C(Nn1cnnc1)c1ccccc1-c1ccccc1 |
| C2.21 | 75042903 | Cc1cnn(Cc2ccccc2Cl)c1NC(=O)c1ccno1 |

| | | |
|---|---|---|
| C2.22 | 6066350 | N=c1sc(Cc2ccccc2Cl)cn1C=C(O)c1ccccc1 |
| C2.23 | 6674418 | O=c1oc2ccccc2c2c1ncn2CCN1CCCCC1 |
| C2.24 | 62962906 | FC1(F)CCN(c2ncnc3c2Cc2ccccc2OC3)CC1 |
| C2.25 | 52847085 | c1ccc2c(c1)Cn1c(nnc1C1CCOCC1)CO2 |
| C2.26 | 82651675 | Cc1n[nH]c(C)c1CCc1nnc2n1Cc1ccccc1OC2 |
| C2.27 | 40336121 | CSCCN(C)c1ncnc2c1Cc1ccccc1OC2 |
| C2.28 | 22473152 | c1ccc2c(c1)Cn1c(nnc1[C@@H]1CCCN1)CO2 |
| C2.29 | 6589314 | N1(C(=O)c2cnccc2)c2c(CCc3c1cccc3)cccc2 |
| C2.30 | 5230616 | CN(C)CCCC1(O)c2ccccc2CCc2ccccc21 |
| C2.31 | 97204205 | O=C1CC(c2nnc3n2Cc2ccccc2OC3)CN1Cc1ccccc1 |
| C2.32 | 33797769 | c1ccc2c(c1)Cn1c(nnc1Cn1cccn1)CO2 |
| C2.33 | 86802957 | Cn1ccnc1NC(=O)c1ccccc1-n1cccn1 |
| C2.34 | 15485822 | c1ccc2c(c1)Cn1c(nnc1-c1ccncc1)CO2 |
| C2.35 | 10496092 | Cn1ccnc1-c1nnc2n1Cc1ccccc1OC2 |
| C2.37 | 22393981 | OCC1CCCN(c2ncnc3c2Cc2ccccc2OC3)C1 |
| C2.38 | 47102716 | c1cncc(Cc2nnc3n2Cc2ccccc2OC3)c1 |
| C2.39 | 47933232 | c1ccc2c(c1)Cn1c(CCn3cccn3)nnc1CO2 |
| C2.40 | 74172808 | Oc1ccccc1CCc1nnc2n1Cc1ccccc1OC2 |
| C2.41 | 45434360 | c1ccc2c(c1)Cc1c([nH]cnc1=NCC1CC1)CO2 |
| C2.42 | 83861598 | CN(Cc1ncc[nH]1)c1ncnc2c1Cc1ccccc1OC2 |
| C2.43 | 32684430 | c1ccc(NCCc2nnc3n2Cc2ccccc2OC3)cc1 |
| C2.44 | 41687630 | Cc1nc2c(c(N[C@@H](CO)C(N)=O)n1)Cc1ccccc1OC2 |
| C2.45 | 40347695 | CN(C)S(=O)(=O)CCNc1ncnc2c1Cc1ccccc1OC2 |
| C2.46 | 58189388 | CN(C)CCN(C)c1ncnc2c1Cc1ccccc1OC2 |
| C2.47 | 29407967 | c1ccc2c(c1)Cc1c(ncnc1NCCN1CCCC1)CO2 |
| C2.48 | 94539214 | Cc1nc2c(c(N(C)C)n1)Cc1ccccc1OC2 |
| C2.49 | 76479036 | Cc1cnn(Cc2ccccc2Cl)c1NC(=O)c1ncn[nH]1 |
| C2.50 | 42815804 | COCCn1cncc1CNc1ncnc2c1Cc1ccccc1OC2 |
| C2.51 | 25919507 | Cc1nc(=NCCCc2ncc[nH]2)c2c([nH]1)COc1ccccc1C2 |
| oxcarbazepine | N/A | C1C2=CC=CC=C2N(C3=CC=CC=C3C1=O)C(=O)N |
| eslicarbazepine acetate | N/A | CC(=O)O[C@H]1CC2=CC=CC=C2N(C3=CC=CC=C13)C(=O)N |
| tianeptine | N/A | CN1C2=CC=CC=C2C(C3=C(S1(=O)=O)C=C(C=C3)Cl)NCCCCCCC(=O)O |

Source Data for Supplementary Information:

Supplementary Figure 10B:

SDS-PAGE gel of DrOTOP1 nanodisc sample size-exclusion chromatography (SEC) purification.

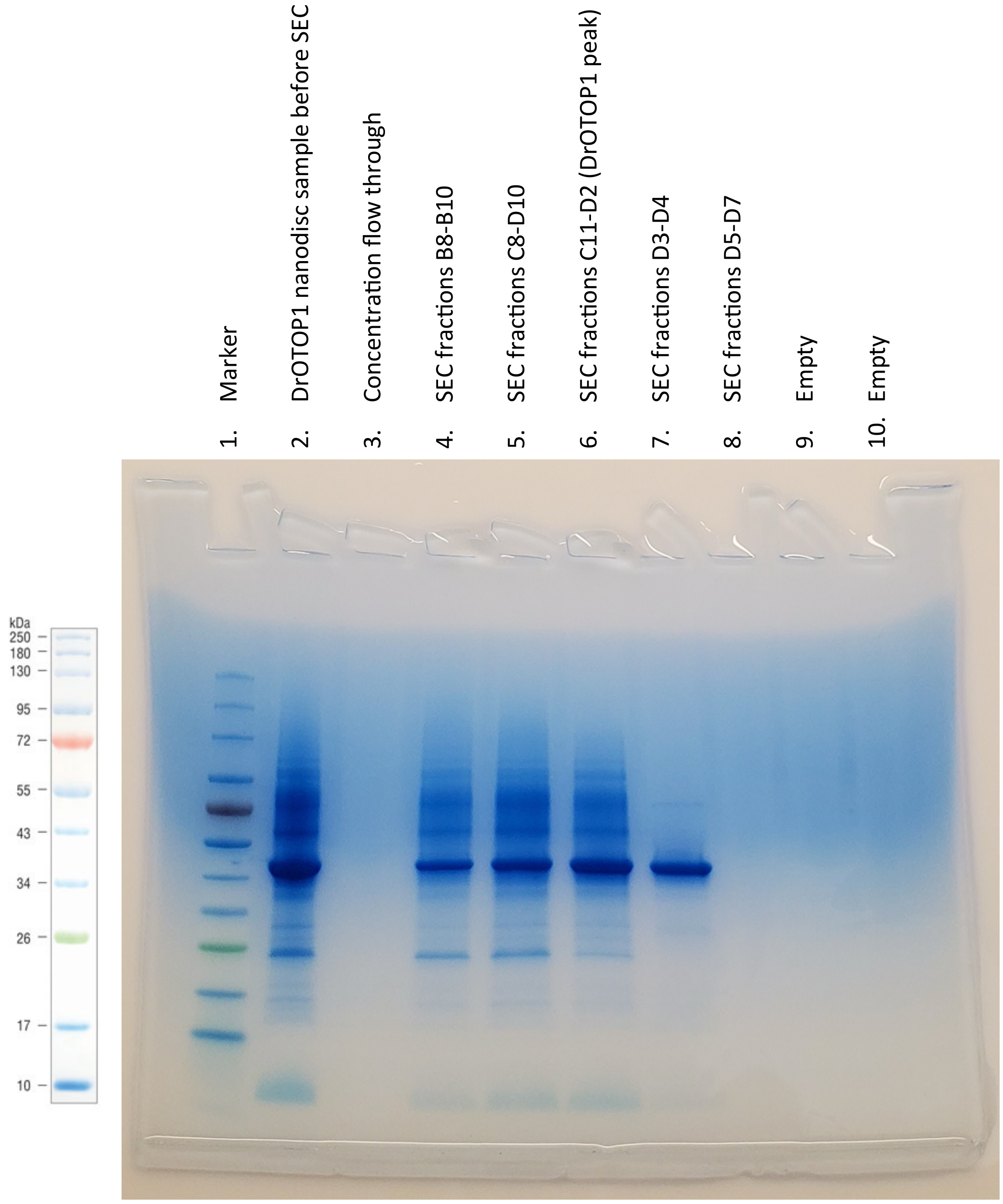

Supplementary Figure 11B:

SDS-PAGE gel of DrOTOP1 nanodisc sample SEC purification.

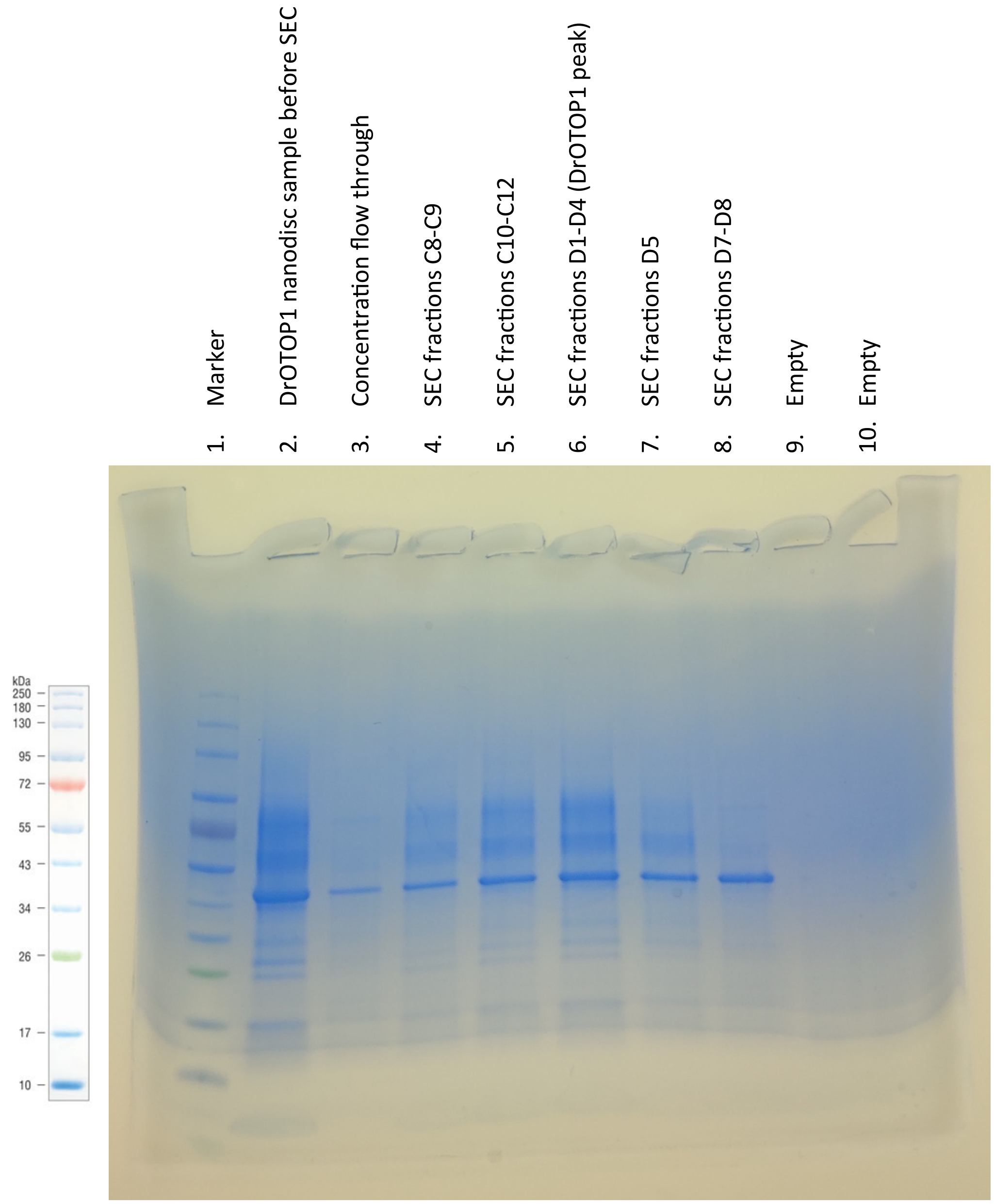

Supplementary Figure 11H:

SDS-PAGE gel of DrOTOP1 nanodisc sample SEC purification.

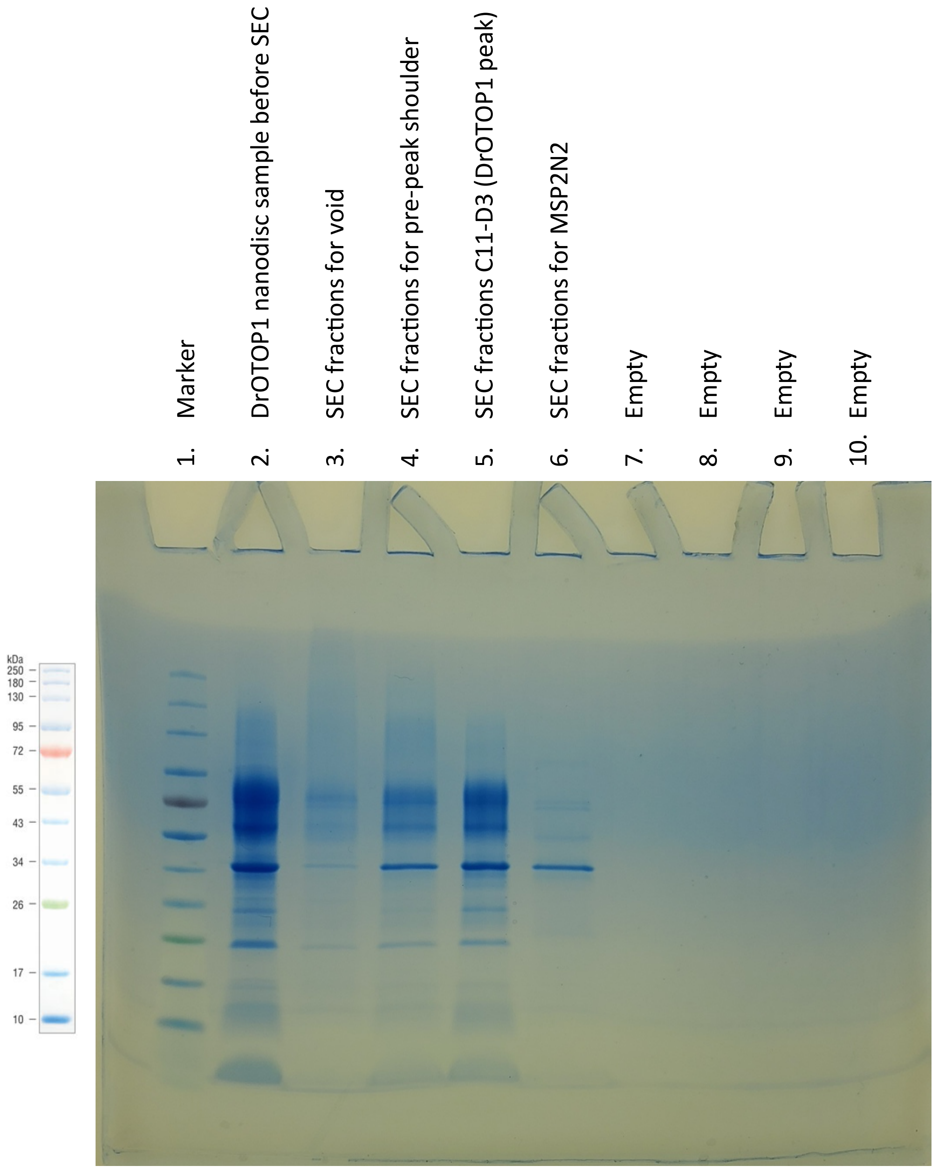

Supplement: Supplementary file 1 — Supplementary information [file 41467_2025_64392_MOESM1_ESM.pdf]
