# Supplementary material for: Structure-guided discovery of Otopetrin 1 inhibitors reveals druggable binding sites at the intrasubunit interface
Source: Nat Commun. 2025 Oct 23;16:9362. doi: 10.1038/s41467-025-64392-0 (PMC12549899; doi:10.1038/s41467-025-64392-0)
Supplement: Supplementary file 2 — Description of Additional Supplementary Files [file 41467_2025_64392_MOESM2_ESM.pdf]

Title: Supplementary Movie 1

Description: Conformational difference between DrOTOP1_apo and DrOTOP1_C2.2 structures Movie of a morph between the DrOTOP1_apo (green and light green cartoons), representing the apo state and DrOTOP1_C2.2 models (purple and pink cartoons) representing the inhibitor C2.2 bound state, with two copies of C2.2 bound (cyan spheres) in the outer and central sites. N-domains are colored darker shades and C-domains colored lighter shades. 0-6 s: Side view of DrOTOP1_apo, showing the outer site. 6-11 s: Morph from apo state to C2.2 bound state. 11-22 s: 180º degree rotation view of both models, ending view showing the central site. 22-28 s: Morph from the C2.2 bound state to the apo state and back. 28-37 s: 180º degree rotation view back to a side view of the outer site. 37-40 s: 90º rotation from side view to bottom (intracellular) view. 40-47 s: Morph between the two states, highlighting shifts in the intracellular ends of TM helices near binding site. Illustrated using ChimeraX.
