# Supplementary material for: Structure-guided discovery of Otopetrin 1 inhibitors reveals druggable binding sites at the intrasubunit interface
Source: Nat Commun. 2025 Oct 23;16:9362. doi: 10.1038/s41467-025-64392-0 (PMC12549899; doi:10.1038/s41467-025-64392-0)
Supplement: Supplementary file 4 — Reporting Summary [file 41467_2025_64392_MOESM4_ESM.pdf]

nature portfolio

Corresponding author(s): Liman, ER
Forli, S
Ward, AB

Last updated by author(s): Aug 26, 2025

Reporting Summary

Nature Portfolio wishes to improve the reproducibility of the work that we publish. This form provides structure for consistency and transparency in reporting. For further information on Nature Portfolio policies, see our Editorial Policies and the Editorial Policy Checklist.

Statistics

For all statistical analyses, confirm that the following items are present in the figure legend, table legend, main text, or Methods section.

n/a	Confirmed	
☐	☒	The exact sample size (*n*) for each experimental group/condition, given as a discrete number and unit of measurement
☐	☒	A statement on whether measurements were taken from distinct samples or whether the same sample was measured repeatedly
☐	☒	The statistical test(s) used AND whether they are one- or two-sided<br>*Only common tests should be described solely by name; describe more complex techniques in the Methods section.*
☒	☐	A description of all covariates tested
☐	☒	A description of any assumptions or corrections, such as tests of normality and adjustment for multiple comparisons
☐	☒	A full description of the statistical parameters including central tendency (e.g. means) or other basic estimates (e.g. regression coefficient) AND variation (e.g. standard deviation) or associated estimates of uncertainty (e.g. confidence intervals)
☐	☒	For null hypothesis testing, the test statistic (e.g. *F*, *t*, *r*) with confidence intervals, effect sizes, degrees of freedom and *P* value noted<br>*Give P values as exact values whenever suitable.*
☒	☐	For Bayesian analysis, information on the choice of priors and Markov chain Monte Carlo settings
☒	☐	For hierarchical and complex designs, identification of the appropriate level for tests and full reporting of outcomes
☒	☐	Estimates of effect sizes (e.g. Cohen's *d*, Pearson's *r*), indicating how they were calculated

Our web collection on statistics for biologists contains articles on many of the points above.

Software and code

Policy information about availability of computer code

Data collection	AutoDock Vina v1.1.2 software, AutoDock-GPU v1.6, AutoGrid v4.2.6, and python code packages RDKit v2021.09.3, molscrub v0.1.0, and Meeko v0.6.1 were used for generation of molecular docking data.<br>AutoDock Vina v1.1.2 software is available at https://vina.scripps.edu<br>AutoDock-GPU v1.6 is available on GitHub at https://github.com/ccsb-scripps/AutoDock-GPU<br>AutoGrid v4.2.6 is available on GitHub at https://github.com/ccsb-scripps/autogrid<br>RDKit v2021.09.3 is available at https://www.rdkit.org<br>molscrub v0.1.0 and Meeko v0.6.1 are available on GitHub at https://github.com/forlilab<br><br>Leginon and EPU 2 softwares were used for automated collection of EM data.<br>pClamp 8.2 software was used for collection of whole-cell patch clamp data.
Data analysis	For virtual screening and molecular docking, initial filtering of docked molecules were done using scripts from Raccoon2, from the AutoDock suite and MGL Tools v1.5.7. All downstream analysis was done using RDKit v2021.09.3, UMAP v0.5.9, and HDBSCAN v0.8.39 python code packages. Final visual inspection was done in PyMOL v2.5.<br><br>For cryo-EM data analysis, micrographs were aligned and dose-weighted using MotionCor2 and Patch Motion Correction in cryoSPARC Live v4.4.1, v3.3.0, and v4.1.1. CTF estimation was done using CTFFIND4 and Gctf, as wrappers inside cryoSPARC, with corresponding versions. All following processing was done in cryoSPARC. All model building and real-space refinement steps were performed using Coot v0.9.8.7, Phenix v1.20.1 and Rosetta v2022.11. Small molecule refinement parameters were obtained from eLBOW in Phenix v1.20.1. Structural figures were made using UCSF ChimeraX v1.10 and PyMOL v2.5.

For electrophysiology data analysis, recordings were analyzed using Clampfit 10. Electrophysiological statistical analysis and graphs were made using GraphPad Prism 10.

For manuscripts utilizing custom algorithms or software that are central to the research but not yet described in published literature, software must be made available to editors and reviewers. We strongly encourage code deposition in a community repository (e.g. GitHub). See the Nature Portfolio guidelines for submitting code & software for further information.

Data

Policy information about availability of data

All manuscripts must include a data availability statement. This statement should provide the following information, where applicable:

- Accession codes, unique identifiers, or web links for publicly available datasets
- A description of any restrictions on data availability
- For clinical datasets or third party data, please ensure that the statement adheres to our policy

Small molecules reported in the manuscript are available from ChemBridge (http://www.hit2lead.com/), searchable by their Chembridge IDs listed in Supplementary Table 1 in the manuscript.
3D maps and models from the EM analysis have been deposited to the Electron Microscopy Databank (http://www.emdatabank.org/) and the Protein Data Bank (http://www.rcsb.org/), respectively. The accession numbers are listed in the Data availability section and Table 2 included in the manuscript.

Research involving human participants, their data, or biological material

Policy information about studies with human participants or human data. See also policy information about sex, gender (identity/presentation), and sexual orientation and race, ethnicity and racism.

Reporting on sex and gender	N/A
Reporting on race, ethnicity, or other socially relevant groupings	N/A
Population characteristics	N/A
Recruitment	N/A
Ethics oversight	N/A

Note that full information on the approval of the study protocol must also be provided in the manuscript.

Field-specific reporting

Please select the one below that is the best fit for your research. If you are not sure, read the appropriate sections before making your selection.

☒ Life sciences ☐ Behavioural & social sciences ☐ Ecological, evolutionary & environmental sciences

For a reference copy of the document with all sections, see nature.com/documents/nr-reporting-summary-flat.pdf

Life sciences study design

All studies must disclose on these points even when the disclosure is negative.

Sample size	Sample size was determined to be consistent with the standard for the field. For electrophysiological recordings from transfected single HEK-293 cells, we always obtained >3 independent separate cells. (Tu et al., 2018, Teng and Kaplan et al. 2023, Liang et al. 2023).
Data exclusions	We measured currents of all DrOTOP1 mutations (Supplementary Figs. 2 and 5), in response to an 8 s pH 5.5 stimulus and excluded mutants with currents below 25 pA from further analysis and study.
Replication	All replicates from independent cells are included in the data, as indicated by n in each figure legend. The data were consistently reproducible.
Randomization	Randomization is not applicable to our study since all tested constructs belong to a group defined by DNA sequence.
Blinding	Blinding is not relevant for patch clamp recordings. Each experiment included controls, and all data were automatically recorded to prevent potential investigator bias from influencing the outcome.

Reporting for specific materials, systems and methods

We require information from authors about some types of materials, experimental systems and methods used in many studies. Here, indicate whether each material, system or method listed is relevant to your study. If you are not sure if a list item applies to your research, read the appropriate section before selecting a response.

Materials & experimental systems

n/a	Involved in the study
☒	☐ Antibodies
☐	☒ Eukaryotic cell lines
☒	☐ Palaeontology and archaeology
☒	☐ Animals and other organisms
☒	☐ Clinical data
☒	☐ Dual use research of concern
☒	☐ Plants

Methods

n/a	Involved in the study
☒	☐ ChIP-seq
☒	☐ Flow cytometry
☒	☐ MRI-based neuroimaging

Eukaryotic cell lines

Policy information about cell lines and Sex and Gender in Research

Cell line source(s)	HEK-293F and HEK-293 (ATCC CRL-153) cells
Authentication	not authenticated
Mycoplasma contamination	HEK-293F lines are tested monthly for mycoplasma. HEK-293 cell health was assessed through visual inspection and viability checks during patch-clamp recordings. To maintain cell line viability, new cells were thawed as needed or at least every 8–12 months.
Commonly misidentified lines (See ICLAC register)	N/A

Plants

Seed stocks	N/A
Novel plant genotypes	N/A
Authentication	N/A
